# Isolation, Characterization, and Comparative Genomic Analysis of Bacteriophage Ec_MI-02 from Pigeon Feces Infecting *Escherichia coli* O157:H7

**DOI:** 10.3390/ijms24119506

**Published:** 2023-05-30

**Authors:** Mohamad Ismail Sultan-Alolama, Amr Amin, Ranjit Vijayan, Khaled A. El-Tarabily

**Affiliations:** 1 Zayed Complex for Herbal Research and Traditional Medicine, Research and Innovation Center, Department of Health, Abu Dhabi 5674, United Arab Emirates; 2 Department of Biology, College of Science, United Arab Emirates University, Al Ain P.O. Box 15551, United Arab Emirates; 3 The Big Data Analytics Center, United Arab Emirates University, Al Ain P.O. Box 15551, United Arab Emirates; 4 Zayed Center for Health Sciences, United Arab Emirates University, Al Ain P.O. Box 17666, United Arab Emirates; 5 Khalifa Center for Genetic Engineering and Biotechnology, United Arab Emirates University, Al Ain P.O. Box 15551, United Arab Emirates; 6 Harry Butler Institute, Murdoch University, Murdoch, WA 6150, Australia

**Keywords:** bacteriophage, bio-preservative, characterization, *E. coli* O157:H7, lysis, phage genome, phage therapy, multiplicity of infection

## Abstract

The most significant serotype of Shiga-toxigenic *Escherichia coli* that causes foodborne illnesses is *Escherichia coli* O157:H7. Elimination of *E. coli* O157:H7 during food processing and storage is a possible solution. Bacteriophages have a significant impact on bacterial populations in nature due to their ability to lyse their bacterial host. In the current study, a virulent bacteriophage, Ec_MI-02, was isolated from the feces of a wild pigeon in the United Arab Emirates (UAE) for potential future use as a bio-preservative or in phage therapy. Using a spot test and an efficiency of plating analysis, Ec_MI-02 was found to infect in addition to the propagation host, *E. coli* O157:H7 NCTC 12900, five different serotypes of *E. coli* O157:H7 (three clinical samples from infected patients, one from contaminated green salad, and one from contaminated ground beef). Based on morphology and genome analysis, Ec_MI-02 belongs to the genus Tequatrovirus under the order Caudovirales. The adsorption rate constant (K) of Ec_MI-02 was found to be 1.55 × 10^−8^ mL/min. The latent period was 50 min with a burst size of almost 10 plaque forming units (pfu)/host cell in the one-step growth curve when the phage Ec_MI-02 was cultivated using the propagation host *E. coli* O157:H7 NCTC 12900. Ec_MI-02 was found to be stable at a wide range of pH, temperature, and commonly used laboratory disinfectants. Its genome is 165,454 bp long with a GC content of 35.5% and encodes 266 protein coding genes. Ec_MI-02 has genes encoding for rI, rII, and rIII lysis inhibition proteins, which supports the observation of delayed lysis in the one-step growth curve. The current study provides additional evidence that wild birds could also be a good natural reservoir for bacteriophages that do not carry antibiotic resistance genes and could be good candidates for phage therapy. In addition, studying the genetic makeup of bacteriophages that infect human pathogens is crucial for ensuring their safe usage in the food industry.

## 1. Introduction

*Escherichia coli* was first discovered in 1885 by Theodor Escherich [1]. Enterohemorrhagic *E. coli*, also known as EHEC, is a subset of Shiga toxin-producing *E. coli* (STEC), and it has recently been identified as one of the principal foodborne pathogens [2]. *E. coli* O157:H7 is the most important serotype of STEC [3] for its role in causing foodborne illnesses [2,3]. *E. coli* O157:H7 has a low infectious dose of 50–100 colony-forming units (cfu)/g of solid materials or 50–100 cfu/mL of liquid materials [4] due to its stress resistance mechanisms [4] and surviving in low pH environments such as acidic food [5]. Survival in the stomach’s acidic environment is critical for the pathogen before it can colonize the small intestine and/or colon [5,6].

*E. coli* O157:H7 can cause various gastroenteritis symptoms such as diarrhea [7,8], hemolytic uremic syndrome [2,9], hemorrhagic colitis [10], and thrombotic thrombocytopenic purpura [11], and may even cause death [12]. In fact, recently the Centers for Disease Control and Prevention (CDC) in the USA reported two *E. coli* O157:H7 outbreaks in September 2022; one outbreak was linked to ground beef [13] and the other was due to an unknown food source [14]. The main sources of *E. coli* O157:H7 infection are poultry and their products [15], livestock [16], polluted water [17], and leafy vegetables [17,18]. 

Furthermore, multidrug-resistant *E. coli* is a global health challenge in human and veterinary medicine [19]. Different pathogenic and commensal *E. coli* have successfully developed antibacterial resistance by acquiring, for example, genes that encode for carbapenemases (becoming resistant to carbapenems), plasmid-mediated quinolone resistance (PMQR) (becoming resistant to [fluoro]quinolones), extended-spectrum β-lactamases (becoming resistant to cephalosporins), mobile colistin resistance (becoming resistant to polymyxins), and 16S rRNA methylases (becoming resistant to aminoglycosides) [19].

Traditional food preservation techniques have been used to reduce harmful bacteria in food, such as *E. coli* O157:H7 [20,21]. These include pasteurization, radiation, food preservatives, and lactic acid bacteria [20,21,22]. However, the effectiveness of these procedures varies according to the inherent features of the foods, and they may also alter the sensory attributes of the finished product [21]. In this context, several strategies are presented as potential solutions for controlling biological contamination in foods [23,24]. For instance, recent research has recommended the use of bacteriophages to regulate bacteria for the purpose of food production and processing [23], and the outcomes of these investigations have shown promise [24,25]. The United States Food and Drug Administration (FDA) approved the use of phages as food preservatives in 2006. Subsequently, several phage products were developed as safe bacterial biocontrol agents [25,26].

ListShield™ (used in salami, sausage, pastrami, seafood, food contact surfaces, and settings to control *Listeria monocytogenes*) is one such product [25,26]. Other commercially available phage products include SalmoFresh™ (applied to poultry, fish and shellfish, fresh and processed fruits, and vegetables to control *Salmonella enterica*) [26] and EcoShield^TM^ (applied to red meat parts and trim intended to be grounded to control *E. coli* O157:H7) [26]. All these phage products are made by Intralytix, Inc., which is located in Columbia, MD, USA [26]. In addition, Food Safety in Wageningen, the Netherlands produces PhageGuard Listex to reduce *L. monocytogenes* in ready to eat (RTE) meat and poultry, fish and seafood, and dairy products [26]. AgriPhage^TM^ is also an agricultural bactericide manufactured by OmniLytics Inc. in Sandy, UT, USA for use in agriculture on fruits and vegetables [26].

Bacteriophages are the most common type of infectious agent, and they can be found in every environment on Earth [27,28]. Phages are mainly divided into two categories, virulent phages that replicate via the lytic cycle, and temperate phages that replicate via either the lytic cycle or lysogenic cycle [29]. Virulent phages are widely used in phage therapy, as alternative antibacterial agents, and in the food industry, as a food preservative [25,26,30]. Both these applications are well documented [30]. 

When compared with more conventional techniques, using bacteriophages as antibacterial agents in the food business has some benefits: (i) phages are extremely host-specific, infecting species or even strains within a species that are closely related to one another [31,32]; (ii) phages have never been documented to infect humans or other eukaryotes [33]; (iii) they have little effect on the gut microbiota [33]; (iv) phages do not change the sensory qualities of foods since the genomes of phages do not code for chemicals that can change the color, flavor, or texture of foods [23]; (v) the frequency of phage mutation is higher than that of bacteria, which reduces the likelihood of bacterial resistance [34]; (vi) if their bacterial host is present, phages are capable of self-replication and self-limitation [35]; and (vii) regarding the expense of manufacturing, phages are simple to isolate and cultivate in laboratories [36].

Phage therapy, the use of bacteriophages as a natural antibacterial agent, was introduced soon after the discovery of bacteriophages by Frederick Twort in 1915 [37] and Félix d’Hérelle in 1917 [38]. Bacteriophages, in addition to their use as an alternative natural antibiotic, have many applications in different fields such as phage-display technology [39,40,41], drug-delivery systems [42], diagnostics [43], the food industry, and in environmental sciences [26,30,44,45]. 

Despite several advantages of phage therapy over antibiotics [46,47], phage therapy also has some challenges such as the ability of the bacterial host to develop resistance to its phage [48,49] through many mechanisms and strategies such as preventing phage entry [50,51,52], restriction-modification systems [53], interference with the assembly of bacteriophages [54], CRISPR-Cas systems [55], abortive infection, and toxin-antitoxin systems [56,57] and prokaryotic argonauts [58] that are developed by bacteria to eliminate their attackers. The host specificity of phages or, in other words, the narrow host range of phages is another challenge that limits the efficacy of phages to combat the target bacteria [56,57]. 

There has been an increase in clinical trials of therapeutic phage products as well as an increase in globally distributed phage-based therapy centers [59]. Therefore, the search for new sources for bacteriophages and the isolation and characterization of virulent phages that are capable of eliminating undesired pathogenic bacteria as well as food-contaminating bacteria is in high demand and an urgent necessity [26,29,30,59,60]. 

Humans and animals harbor a wide range of bacteria and, consequently, bacteriophages. This current study suggests that wild animals, specifically wild birds, can be a very good source for bacteriophages. In the current study, we explored wild pigeons and isolated the bacteriophage Ec_MI-02 from its feces in the United Arab Emirates (UAE). Ec_MI-02 was characterized and its genome was sequenced and compared with other *E. coli* phages. The characterization was performed using *E. coli* O157:H7 as the propagation host. 

Ec_MI-02 was the second *E. coli* O157:H7 phage, also isolated from wild pigeons. Previously, we reported UAE_MI-01 [61,62], another *E. coli* O157:H7 phage, from wild pigeons [61,62]. Both phages were characterized to emphasize the diversity of phages within a single source. Thus, this study enriches the collection of phages that can be used to develop phage cocktails for better food preservation and therapeutic applications. 

## 2. Results

### 2.1. Isolation and Host Range of the Phage Ec_MI-02 Active against E. coli O157:H7 Using a Spot Test and an Efficiency of Plating (EOP) Test

The *E. coli* O157:H7 NCTC 12900 bacteriophage, designated Ec_MI-02, was isolated from the droppings of a single wild pigeon nest. Table 1 shows the stock bacterial culture collection that was used to investigate the Ec_MI-02 host range using a spot test. A wide variety of bacterial strains, including closely related *E. coli* as well as other Gram-negative bacteria, were employed in the laboratory evaluation of the Ec_MI-02 host range. Although it is generally accepted that coliphages are unable to infect Gram-positive bacteria, other species of Gram-positive bacteria, including *Staphylococcus*, *Streptococcus*, *Mycobacterium*, *Bacillus*, and *Enterococcus*, have been studied to see whether or not they could serve as hosts for Ec_MI-02 (Table 1). On the bacterial lawn, the presence of plaques was viewed as a positive reaction, whilst the lack of plaques was regarded as a negative reaction.

Ec_MI-02 was identified to infect *E. coli* O157:H7 NCTC 12900 as well as five other serotypes of *E. coli* O157:H7 (three from infected patients, one from contaminated green salad, and one from contaminated ground beef), as well as *E. coli* ATCC 8739 using a spot test. On these hosts, plaques with clear zones were produced by Ec_MI-02. On the other hand, Ec_MI-02 produced turbid plaques on lawns of *E. coli* ATCC 35218, *E. coli* NCTC 13841, *E. coli* ATCC 15223, *E. coli* ATCC 23227, *E. coli* ATCC 9637, *E. coli* ATCC 23224, and an *E. coli* strain isolated from a patient with a urinary tract infection (Table 1). It is noteworthy that the phage Ec_MI-02 was not able to infect other Gram-negative bacteria tested, including *E. coli* ATCC 25922 and an extended-spectrum beta-lactamase (ESBL)-producing *E. coli* from patient blood (Table 1). In addition to this, the phage Ec_MI-02 was not successful in infecting any of the Gram-positive bacteria that were tested (Table 1). 

On the other hand, there was a discrepancy between the results of the host range experiments employing a spot test (Table 1) and an EOP test (Table 2). The EOP test revealed that *E. coli* ATCC 8739, and the five *E. coli* O157:H7 (three from infected patients, one from green salad, and one from ground beef), showed high productive infection (EOP ≥ 0.5), while *E. coli* ATCC NCTC 13841, *E. coli* ATCC 15223, *E. coli* ATCC 23227, and *E. coli* ATCC 9637 had low productive infection (0.001 < EOP < 0.1) compared with the propagation host *E. coli* O157:H7 NCTC 12900. When the EOP test was performed on *E. coli* ATCC 35218, *E. coli* ATCC 23224, and *E. coli* (patient isolate—urine), there was no evidence of plaque development (EOP ≤ 0.001) (Table 2).

These results may explain the failure of some phages in phage therapy. Therefore, *E. coli* O157:H7 NCTC 12900 was used as the propagation host in this study for the characterization experiments. Additionally, based on the genome sequence, HostPhinder, a tool to predict the host cell of a given phage [63], predicted Ec_MI-02 as an *E. coli* phage. 

### 2.2. Plaque Size and Morphology of the Phage Ec_MI-02

**Figure 1:**
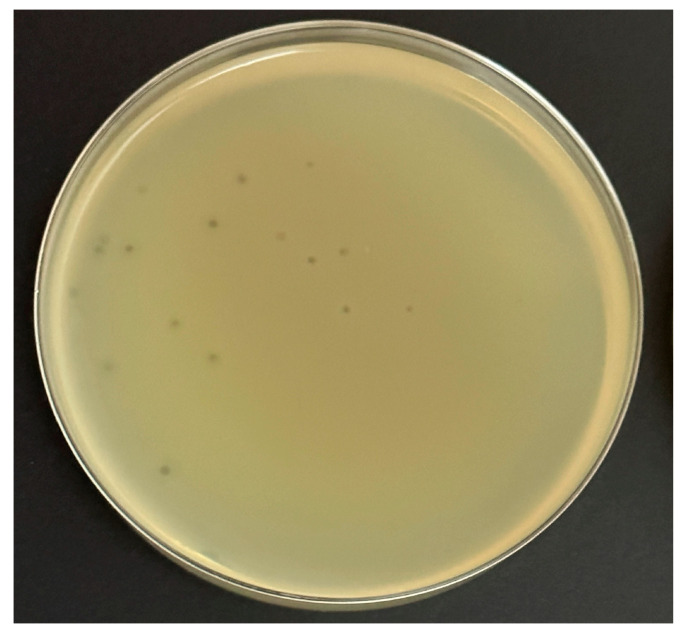
The formation of plaques of the phage Ec_MI-02 on Luria–Bertani agar using *Escherichia coli* O157:H7 NCTC 12900 as the propagation host.

This experiment was performed on four common bacteriological media with a wide range of prices to determine the potential cost of production for any future application. Ec_MI-02 suspensions with 10^2^ plaque forming units (pfu)/mL were used in the plate lysis assay. When *E. coli* O157:H7 NCTC 12900 was used as the propagation host, the Ec_MI-02 phage produced uniform small clear plaques (1–2 mm in size) (Figure 1) that were characteristic of a virulent phage on the following four bacteriological media, Mueller–Hinton agar, tryptone soy bean agar, Luria–Bertani agar, and nutrient agar. There were no significant (*p* > 0.05) differences among the plaque sizes when these four media were used. 

### 2.3. Effect of pH and Temperature on the Phage Ec_MI-02

The pH level is an extremely important factor in all types of food. In phage therapy, the stability of the phage in different pH settings is a significant factor, particularly when the phage is taken orally. Similarly, many foods have different temperature needs for different stages of processing and preservation. In light of this, an investigation of the stability of Ec_MI-02 at a range of pH values (Table 3) and temperatures (Table 4) was carried out. 

The current results indicated that there were no significant differences (*p* > 0.05) found in the log_10_ pfu/mL readings across any of the pH ranges that were investigated (pH 3–10) (Table 3).

Ec_MI-02’s stability in temperatures ranging from −20 °C to 100 °C was tested and the phage was found to be stable up to 65 °C (Table 4). The phage Ec_MI-02 was rendered entirely inactive by being heated for 15 and 30 min at temperatures of 75 °C and 100 °C, respectively (Table 4). There were significant (*p* < 0.05) differences in the log_10_ pfu/mL in all the tested temperature ranges (Table 4). Exposure of phage Ec_MI-02 to temperatures ranging from 25 °C to 65 °C for 15 or 30 min resulted in a significant (*p* < 0.05) decrease in the log_10_ pfu/mL count (Table 4). In addition, 4 °C was shown to be the most stable temperature for Ec_MI-02 compared with −20 °C and 25 °C after 5 months of storage.

### 2.4. Effects of Chemical Disinfectants on the Phage Ec_MI-02

The sensitivity of Ec_MI-02 to three different disinfectants was investigated for a total of 2 min to determine the most effective way to suppress or inactivate the phage in the laboratory. 

Ec_MI-02 was found to be relatively resistant to 70% ethanol. There were significant (*p* < 0.05) differences in the log_10_ pfu/mL when the phage was exposed to ethanol for 60 s compared with 30 s (Table 5). However, there were no significant (*p* > 0.05) differences when the time of exposure increased from 0 to 30 s (Table 5). When the exposure time was increased from 60 to 120 s, however, there were no statistically significant (*p* > 0.05) differences (Table 5).

When the commercial disinfectant at 20% was employed, there were significant variations (*p* < 0.05) in the log_10_ pfu/mL when the length of exposure increased from 30 to 60 to 120 s (Table 5). The commercial disinfectant caused one log reduction after 60 s and two log reductions after 120 s (Table 5).

However, 2% sodium hypochlorite was able to totally inactivate the phage Ec_MI-02 within 30 s (Table 5). According to these data, sodium hypochlorite at a concentration of 2% was appreciably more effective than ethanol at a concentration of 70%, which is the standard concentration utilized in laboratories. 

### 2.5. Adsorption Time, Adsorption Rate Constant, Latent Period, and Burst Size of the Phage Ec_MI-02

To measure the adsorption time, adsorption rate constant, latent period, and burst size, the phage Ec_MI-02 was propagated in the host cell *E. coli* O157:H7 NCTC 12900. Adsorption was observed within 15 min (eclipse period) with 99% efficiency. Ec_MI-02’s adsorption rate constant (K) was determined to be 1.55 × 10^−8^ mL/min.

**Figure 2:**
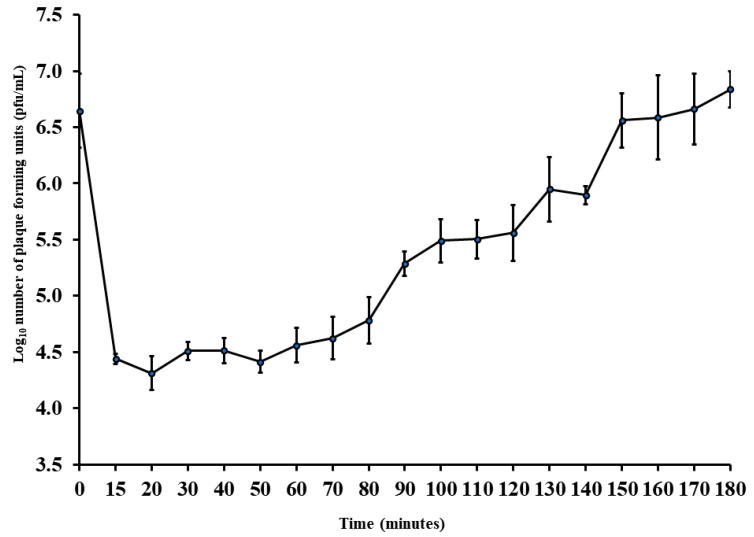
One-step growth curve of the phage Ec_MI-02 with *Escherichia coli* O157:H7 NCTC 12900 as the propagation host. Values are means ± standard deviation of four replicates for each timing. Bars represent standard deviation. pfu = plaque forming units.

The latent period was almost 50 min with a burst size of almost 10 pfu/host cell (Figure 2). As a result, this phage has the potential to be manufactured economically as a bio-preservative in addition to being used for phage therapy.

### 2.6. Transmission Electron Microscopy (TEM) of the Phage Ec_MI-02

**Figure 3:**
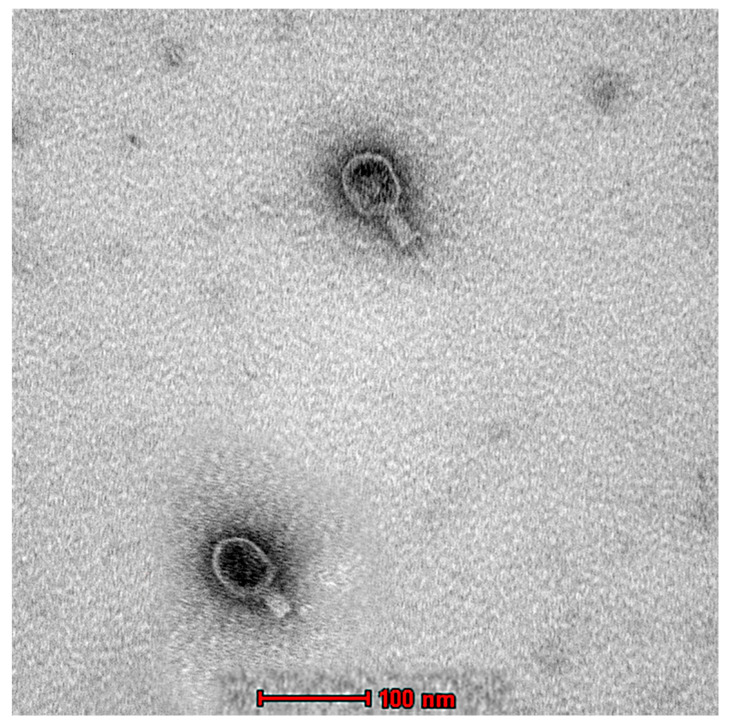
Transmission electron micrograph of the phage EC_MI-02. Scale bar = 100 nm.

The morphological characterization of Ec_MI-02 was studied using TEM, which, along with genomic analysis, indicated that the Ec_MI-02 bacteriophage belongs to the Tequatrovirus genus in the order Caudovirales. The phage was approximately 220 nm long and 80 nm wide with a 100 × 80 nm head and a 120 × 20 nm contractile tail (Figure 3). 

### 2.7. Genomic Analysis and Bioinformatics of the Phage Ec_MI-02

**Figure 4:**
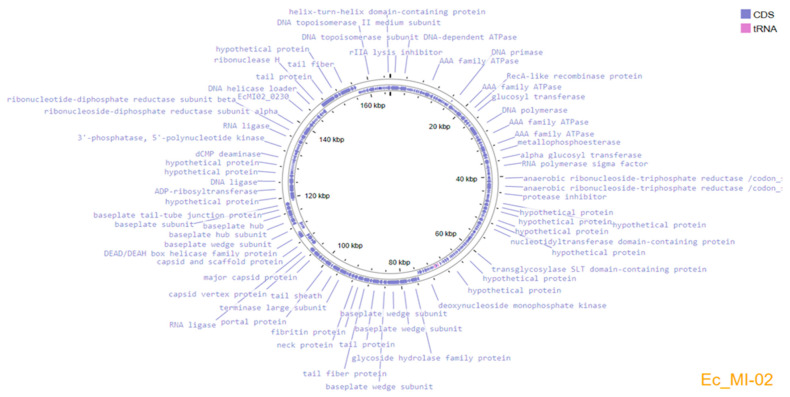
Genome organization map of Ec_MI-02 created with Proksee, showing annotated ORFs.

Genome assembly using the three subsampled reads produced the same assembled genome sequence of 165,454 bp length and a GC content of 35.5%. The complete genome sequence of Ec_MI-02 has been deposited in the NCBI GenBank with accession OP856590. Annotation of the genome predicted 266 protein-coding genes and 9 tRNAs, which covered 94.7% of the genome (Figure 4). The genome encodes structural/assembly genes, including genes for terminase large and small subunits, capsid proteins, assembly proteins, and tail fiber proteins.

Replication/transcription-related genes included DNA helicase, DNA helicase loader, DNA polymerase, DNA topoisomerase, and RNA polymerase. Analysis with ABRicate [64] did not identify any antimicrobial resistance or virulence genes. The presence of holin along with glycoside hydrolase family protein (lysozyme/endolysins) suggests that Ec_MI-02 could be a virulent bacteriophage. No lysogeny-associated genes, such as integrase, excisionase, transposase, etc., were identified in the Ec_MI-02 genome.

**Figure 5:**
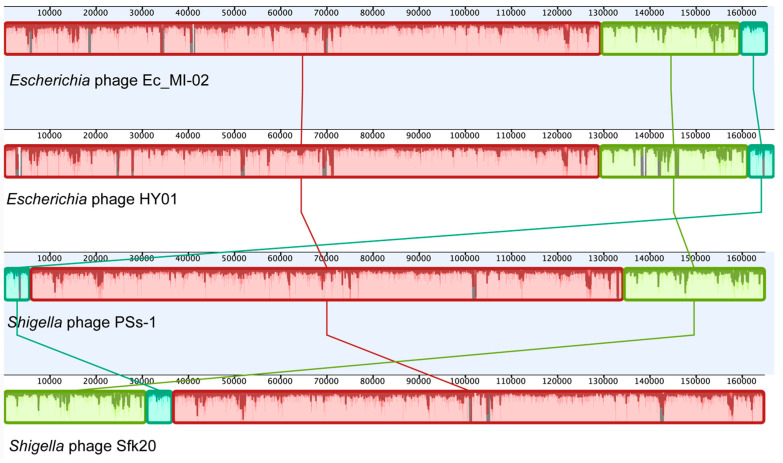
Whole genome alignment of the bacteriophage Ec_MI-02 and the three phages with genome sequences most identical to the genome of Ec_MI-02 identified through a BLASTN search. The alignment was generated using progressiveMauve. Blocks with the same color match regions in the selected genomes.

An NCBI BLASTN search (Table 6) revealed that Ec_MI-02 shared 98.56% sequence identity with *Shigella* phage pSs-1 (97% coverage) with GenBank accession NC_025829.1, 98.44% sequence identity with *Shigella* phage Sfk20 (96% coverage) with GenBank accession MW341595.1, and 97.57% sequence identity with *Escherichia* phage HY01(96% coverage) with GenBank accession KF925357.1. Whole genome alignment of the four bacteriophages (Ec_MI-02, pSs-1, Sfk20, and HY01) with progressiveMauve indicated the conservation of order of the genomic blocks (Figure 5).

**Figure 6:**
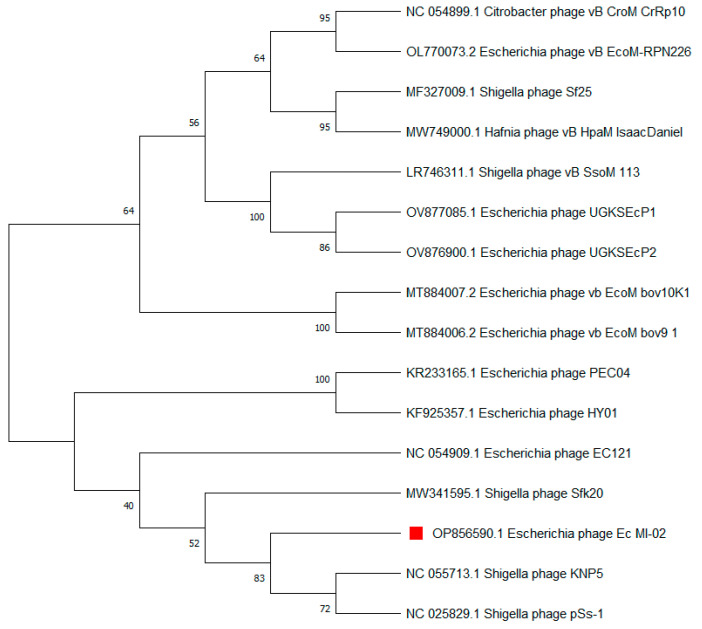
Phylogenetic tree constructed using the terminase large (*TerL*) subunit gene sequences of Ec_MI-02 phage (red square) and 15 closest sequences identified using an NCBI BLASTN search. Sequences were aligned using MUSCLE in MEGA 11 and the phylogenetic tree was generated using the maximum likelihood method with 100 bootstraps.

The terminase large subunit (*TerL*) is generally used as a genetic marker for phages of the order Caudovirales. Here, *TerL* sequences most similar to that of Ec_MI-02 were identified using a nucleotide BLAST (BLASTN) search. Subsequently, a phylogenetic tree was constructed, which placed Ec_MI-02’s *TerL* close to that of *Shigella* phages KNP5 and pSs-1 (Table 6, Figure 6). 

## 3. Discussion

As the prevalence of antibiotic resistant bacteria increases, the demand for new bacteriophages also increases. In order to diversify the range of available phages, new sources should be explored. Wild animals and birds are good sources of phages that are potentially free of drug-resistant genes [64]. In the present study, we examined bird feces for phages of the verotoxin producing *E. coli* O157:H7, an important foodborne pathogen that was responsible for many outbreaks worldwide [12]. *E. coli* O157:H7 infection can cause many pathological symptoms such as abdominal cramps, diarrhea, hemolytic colitis, hemolytic uremic syndrome, and kidney failure, which are often very serious and may lead to death [8,9,10,11,12]. In fact, two separate *E. coli* O157:H7 outbreaks were reported in September 2022 by the CDC in the USA; one was associated with ground beef [13] and the other was caused by an unidentified food source [14].

Bacteriophages can recognize their bacterial hosts and eliminate them. Therefore, bacteriophages are potent bacterial biocontrol agents [23,24,25,26,60]. In the present study, Ec_MI-02, a virulent phage of *E. coli* O157:H7, was isolated from pigeon feces and characterized. The host range investigation using a spot test assay and EOP analysis showed that Ec_MI-02 targets the *E. coli* O157:H7 strain in addition to a few other *E. coli* strains. However, Ec_MI-02 produced turbid plaques and behaved as a prophage in some other *E. coli* strains. Based on Yoichi et al. [65], the virulent phage of a strain could act as a prophage in related strains which do not have fully compatible receptors. This could be due to the ability of some of the related strains to develop resistance to that phage. This could explain the failure of some phages in phage therapy and justify the importance of a phage cocktail [65]. The host range was also supported by the prediction of the in silico HostPhinder tool, which showed that Ec_MI-02 is an *E. coli* bacteriophage [63]. Several previously reported bacteriophages of *E. coli* O157:H7 showed wider or narrower host ranges [64]. 

In the present study, Ec_MI-02 was successfully grown on four different types of media, including Mueller–Hinton agar, tryptone soy bean agar, Luria–Bertani agar, and nutrient agar using *E. coli* O157:H7 NCTC 12900 as the propagation host. Ec_MI-02 was able to produce clear plaques on all four media. Therefore, it is possible to propagate and culture it using any of the four media indicated above, which could have an impact on costs and commercial production [24,25,26].

To identify phage genomes that are close in sequence to Ec_MI-02, NCBI nucleotide BLAST (BLASTN) was employed (Table 6). Only three of the closest ten phages have been characterized—*Shigella* phage pSs-1, isolated from environmental water in South Korea [66]; *Shigella* phage Sfk20, isolated from water bodies of a diarrheal outbreak location in India [67]; and *Escherichia* phage HY01 [68]. Previously, in the UAE, we had reported UAE_MI-01, another *E. coli* phage from the same source with a much smaller genome (44,281 bp) [61,62]. Nonetheless, we included it in the comparison to demonstrate the extent of diversity in phages within the single source (same nest) [62].

Ec_MI-02 was similar in size to pSs-1 (218 × 98 nm) [66], but larger than Sfk20 [67] (190 × 62 nm), HY01 [68] (200 nm long), and UAE_MI-01 [62] (180 × 50 nm). Ec_MI-02 showed higher temperature stability, since it was stable at 65 °C, compared with pSs-1 [66], which was stable only up to 50 °C, and Sfk20 [67] and HY01 [68], which were found to be deactivated at a temperature of around 50 °C. However, Ec_MI-02 was quite similar to UAE_MI-01 [62], both of which could tolerate temperatures up to 65 °C. 

Ec_MI-02 was also found to be stable in the pH range of 3–10, which was similar to HY01 [68], but more stable than pSs-1 [66], with a pH range of 5–9, and Sfk20 [67], with a range of 7–9. Therefore, Ec_MI-02 is an excellent candidate for use as a biocontrol agent in the veterinary, food, and agricultural industries due to its host range in addition to its stability in a wide range of temperatures and pH values. This stability is necessary for surviving during the steps of food processing as well as environmental conditions as suggested by [23,26]. 

Ec_MI-02 has a latent period of almost 50 min (Figure 2), which is longer than UAE_MI-01 (20 min) [62] and the closest bacteriophages that had latent periods of 15 min (HY01) [68], 20 min (Sfk20) [67], and 25 min (pSs-1) [66]. The burst sizes of UAE_MI-01 [62], HY01 [68], pSs-1 [66], and Sfk20 [67] were 100, 100, 97, and 123 pfu/host cell, respectively. However, the result of the one-step growth experiment in the current study for the Ec_MI-02 suggests that there is an irregular delayed lysis with a burst size of almost 10 pfu/host cell. 

Large T4 phages and their relatives are known for a unique type of phage-encoded plasticity that is known as lysis inhibition [69]. Bull et al. [70] stated that not all phages lyse their hosts; for example, fd, f1, and M13 are *E. coli* phages that are released through the host’s cellular membrane without destroying the host cell [70]. Annotation of the Ec_MI-02 genome in the present study revealed that it has genes encoding for *r*I, *r*IIA, and *r*III lysis inhibition proteins. *r*I and *r*III were found to have a direct effect on lysis inhibition. *r*II has an indirect control in the lysis inhibition process [71]. The presence of the genes *r*I, *r*IIA, and *r*III and their direct and indirect roles in delaying cell lysis could explain the pattern of the one-step growth curve in the current study. Nine tRNAs were annotated in the genome. While the precise purpose is unknown, these could be used for codon compensation or perhaps even to compensate for tRNA-depleting techniques used by the host [72].

The two characterized phages UAE_MI-01 [62] and Ec_MI-02 were isolated and identified from the wild pigeon feces. Ec_MI-02 and UAE_MI-01 [61,62] were isolated on the same day from feces of the same nest. They were initially differentiated according to their plaque size. Plaques developed by the UAE_MI-01 on Luria–Bertani agar were larger in size (between 5 and 6 mm in diameter) [62], while plaques produced by the Ec_MI-02 utilizing the same propagation host *E. coli* O157:H7 NCTC 12900 were smaller (between 1 and 2 mm in diameter).

TEM and whole genome sequencing confirmed that they were different phages [62,64]. The two phages are morphologically different (Supplementary Figure S1). UAE_MI-01 [62] belongs to family Siphoviridae in the order Caudovirales, whilst Ec_MI-02 belongs to the genus Tequatrovirus under the order Caudovirales.

In addition, both phages showed different host ranges against *E. coli* (Supplementary Table S1). Ec_MI-02 was found to be more stable than UAE_MI-01 [62] in acidic pH, while both were stable in alkaline pH (Supplementary Table S2). UAE_MI-01 [62] was found to be more heat resistant than Ec_MI-02 (Supplementary Table S3). UAE_MI-01 was more resistant to chemical disinfectants compared with Ec_MI-02 (Supplementary Table S4). Interestingly, similar to UAE_MI-01, ethanol 70% was not able to cause one log reduction of Ec_MI-02 within 120 s (Table 5, Supplementary Table S4). Sodium hypochlorite 2% showed significant antiviral activity against Ec_MI-02 since it caused six log reductions within 30 s (Table 5). 

UAE_MI-01 [62], which was made of a genome size of 44,281 bp, has an eclipse period of 10 min with 99.9% efficiency and an adsorption rate constant of 1.25 × 10^−7^ mL/min (Supplementary Figure S2), while Ec_MI-02 with a genome size of 165,454 bp has an eclipse period of 15 min with 99% efficiency and an adsorption rate constant of 1.55 × 10^−8^ mL/min (Figure 2, Supplementary Figure S2). The latent period of UAE_MI-01 [62] was about 20 min with a burst size of almost 100 pfu/host cell, whilst Ec_MI-02’s latent period was about 50 min with a burst size of almost 10 pfu/host cell (Supplementary Figure S2). Both phages also showed different genomic organization (Supplementary Figure S3). 

The genome of UAE_MI-01 [62], consisting of 44,281 bp, is notably smaller than that of Ec_MI-02, which is 165,454 bp. Consequently, the Ec_MI-02 genome harbors 266 coding sequences compared with the much lower 64 in UAE_MI-01. Interestingly, while no tRNA genes were identified in UAE_MI-01 [62], nine were annotated in Ec_MI-02.

Bacteriophages infecting human pathogens have been considered potential biocontrol agents, and studying their genetic content is essential to their safe use in the food industry. Our previous work [61,62] and the current results showed that the two bacteriophages (UAE_MI-01 and Ec_MI-02) identified from pigeon feces were distinct phages that might be used in the future to combat *E. coli* O157:H7. Both viruses can be used independently in applications such as phage therapy and food bio-preservatives. They could also potentially be combined in a cocktail for future applications. 

## 4. Materials and Methods

### 4.1. Propagation Media

Luria–Bertani broth (LBB) (tryptone 10 g/L, yeast extract 5 g/L, and sodium chloride 10 g/L pH 7.2) (HiMedia, Mumbai, India) was used in all the protocols in the current study [31,62]. Bacterial dilutions from 18 h LBB cultures grown at 37 °C were carried out in phosphate-buffered saline (PBS; Oxoid, Basingstoke, UK). 

The plaque assay was carried out using soft layer agar [23,25], which was made up of LBB in Lambda buffer (6 mmol/L tris pH 7.2; 10 mmol/L Mg(SO_4_)_2_·7H_2_O; 50 mg/L gelatin (HiMedia), and supplemented with 4 g/L agar (HiMedia).

Mueller–Hinton agar (Mast group, Bootle, UK), tryptone soyabean agar (HiMedia), Luria–Bertani agar (HiMedia), and nutrient agar (HiMedia), were used to determine plaque morphology [25,62].

Ec_MI-02 was maintained and diluted in Lambda buffer and stored at 4°C in all the experiments described below [23,31].

### 4.2. Cultivation of Host Bacterium E. coli O157:H7 

*E. coli* O157:H7 NCTC 12900 was used as the propagation host to isolate the phage Ec_MI-02, and it was also used for all the characterization studies outlined below.

As previously described [62], cultures were stored at −20 °C in LBB with 20% glycerol. Before investigation, a stock culture of the propagation host was maintained on Luria–Bertani agar plates [31,62]. 

One loopful of *E. coli* O157:H7 was used to inoculate a 15 mL sterile centrifuge tube with a flat cup (ExtraGene, Taichung City, Taiwan) containing 10 mL of LBB. The tube was then placed in a shaker incubator set to 37 °C and 70 revolutions/min (rpm) (Innova 4000, New Brunswick Scientific, Edison, NJ, USA) [25,31,62]. The slurry of the bird’s excrement was seeded with this bacterial stock solution as described below. 

### 4.3. Isolation, Purification, and Propagation of the Phage Ec_MI-02 

As described previously [62], pigeon feces from a single nest was collected from Abu Dhabi, UAE, and transferred to a sterile beaker (250 mL). The slurry was prepared with sterile distilled deionized water. Cultures of *E. coli* O157:H7 NCTC 12900 (18 h) were used to seed the slurry every 24 h for 96 h. The beaker was incubated in a shaker incubator at 37 °C and 70 rpm. After 96 h, 10 mL of the slurry was transferred into a 15 mL sterile centrifuge tube with a flat cup and centrifuged for 10 min at 12,000 rpm. Millipore membrane syringe filter (Pore size 0.22 µm, Millipore Corporation, New Bedford, MA, USA) was used to filter the supernatant. The filtrate was then diluted in Lambda buffer using the ten-fold serial dilution technique [31,62]. 

A plate lysis procedure [73,74,75] was used to test all the dilutions for the presence of the phage. Briefly, an aliquot (100 μL) of each dilution was added to 100 μL of 24 h culture of *E. coli* O157:H7 NCTC 12900 (in LBB) in a sterile 1.5 mL Eppendorf micro-centrifuge tube (Greiner Bio-One GmbH, Frickenhausen, Germany). The tubes were gently mixed and were then incubated at 37 °C for 10 min to facilitate bacteriophage–host cell attachment [62,74]. The mixture was then transferred from the Eppendorf micro-centrifuge tube to a 10 mL Bijou bottle, and 2 mL of previously melted soft layer Luria–Bertani agar was then added. The content of the bottle was gently mixed by swirling and pouring it over the surface of a plate of Luria–Bertani agar and was allowed to set for 25 min at room temperature before incubating for 24 h at 37 °C [73,75]. 

As the same pigeon feces from our previous study [62] was used, only plaques with smaller diameters (between 1 and 2 mm) were selected for the current study to ensure the detection of a phage different from the UAE_MI-01 phage [62], which has been shown to produce larger plaques (5–6 mm in diameter) on the same medium with the same propagation host *E. coli* O157:H7 NCTC 12900 [62]. Clear small plaques with a diameter between 1 and 2 mm were picked with a scalpel, transferred to an Eppendorf microcentrifuge tube containing 1 mL of Lambda buffer, and mixed gently before filtration through a sterile Millipore membrane syringe filter (0.22 µm). The filtrate was serially diluted and propagated as mentioned above. Plates with nearly confluent plaques were used to prepare enriched phage suspensions by overlaying with 5 mL lambda buffer. Finally, chloroform was added to separate the bacteriophage from the host cells [76]. Titer of the phage stocks in Lambda buffer was calculated using Miles and Misra technique [77]. The phage stock then was stored at 4 °C for future experiments [62,74].

### 4.4. Determination of the Host Range of the Phage Ec_MI-02

The following bacterial strains were used to determine the ability of Ec_MI-02 to infect various Gram-negative and -positive bacteria including: the propagation host, *E. coli* O157:H7 NTCC 12900, three clinical samples of *E. coli* O157:H7 from infected patients, one *E. coli* O157:H7 serotype isolated from contaminated green salad, one *E. coli* O157:H7 serotype isolated from contaminated ground beef, *E. coli* ATCC 8739, *E. coli* ATCC 25922, *E. coli* ATCC 35218, *E. coli* ATCC NCTC 13841, *E. coli* ATCC 15223, *E. coli* ATCC 23227, *E. coli* ATCC 9637, *E. coli* ATCC 23224, *E. coli* (patient isolate—urine), *E. coli* producing ESBL (patient isolate), *Bacillus subtilis* ATCC 6051, *Pseudomonas aeruginosa* ATCC 25668, *P. aeruginosa* ATCC 27853, methicillin-resistant *Staphylococcus aureus* (patient isolate), *S. aureus* ATCC 6358, *S. aureus* ATCC 29213, *Staphylococcus epidermidis* ATCC 12228, *Staphylococcus saprophyticus* ATCC-BAA 750, *Streptococcus pyogenes* ATCC 19615, *Enterococcus faecalis* ATCC 51299, *E. faecalis* (patient isolate), *Enterococcus casseliflavus* (patient isolate), *Enterobacter aerogenes* ATCC 13018, *Enterobacter hormaechei* (patient isolate), *Klebsiella pneumonia* ESBL-producing ATCC 700603, *K. pneumonia* KPC 2 +ve (patient isolate), *Haemophilus influenzae* ATCC 9007, *Stenotrophomonas maltophilia* ATCC 17666, *Salmonella enterica* ATCC 14028, *Salmonella* sp. (patient isolate), *Proteus vulgaris* ATCC 29905, and *Mycobacterium smegmatis* ATCC 607 (Table 1). 

Phage host range was determined by spotting 10 μL of Ec_MI-02 suspension containing 10^10^ pfu/mL onto dry Luria–Bertani agar plates inoculated with all bacterial strains described above (spot test assay). Plates were incubated for 48 h at 37 °C and examined for the formation of plaques [78].

The Ec_MI-02 phage with the broadest bactericide host range in the spot assays was chosen for a more thorough evaluation of productive infection as specified by the EOP [79,80]. Aliquots (100 µL) of each of the bacterial strains of 18 h culture (at 37 °C) and 100 µL of diluted phage lysate (6 dilutions, since the titer of the original stock was 10^6^ pfu/mL) were mixed as in double layer plaque assays in triplicates [80] and the plates were incubated at 37 °C for 24 h. Pfu were counted for each bacterial strain. A lower dilution was then tested to confirm that the EOP was lower than 0.001 after the 10^2^ dilution failed to produce any plaques [80]. The EOP (average pfu on target bacteria/average pfu on host bacteria) and standard deviation for the three replicates were then determined. The average EOP value for the phage–bacterium combination was labeled as “high production” when the ratio was 0.5 or above, or when the productive infection on the target bacteria produced at least 50% of the pfu discovered for the primary host. EOP values greater than 0.1 but less than 0.5 were classified as “medium production” efficiency, while EOP values between 0.001 and 0.1 were categorized as “low production” efficiency. An EOP value of 0.001 or less was considered inefficient [79,80].

### 4.5. Plaque Morphology of the Phage Ec_MI-02

To investigate the ability of the phage Ec_MI-02 to produce uniform plaques on different media, Ec_MI-02 suspension containing 10^2^ pfu/mL was used following the plate lysis procedure described above [78,80]. Four different media were used, namely, Mueller–Hinton agar (Mast group), tryptone soyabean agar (HiMedia), Luria–Bertani agar (HiMedia), and nutrient agar (HiMedia). Ec_MI-02 was grown on all four media to determine which type of these common bacteriological media Ec_MI-02 prefers. This could ultimately determine how much it will cost to commercially produce the phage for any future applications. 

### 4.6. Effect of Physical and Chemical Agents on Stability and Viability of the Phage Ec_MI-02

The impact of a few selected physical and chemical factors on the survival and proliferation of the phage Ec_MI-02 was investigated, according to Brownell et al. [81].

To determine the effects of the selected physical treatments on phage propagation and viability, LBB-grown Ec_MI-02 phage was diluted 1 to 10 in LBB. Sample (0.1 mL) was added to 0.9 mL of LBB, and the physical treatment was applied according to the desired concentration. Viable phage numbers were estimated in the selected time points after the treatment, and they was expressed as pfu/mL. The control used was a similarly diluted Ec_MI-02 phage, but was untreated with the selected physical treatments [81]. 

The physical treatments tested were pH and temperature. To determine the effect of pH on the viability and propagation of the phage Ec_MI-02, different pH values (pH 3, 4, 7, 9, 10) were used. Aliquots (0.1 mL) of phage suspension (10^9^ pfu/mL) were added to 0.9 mL of each pH value in an Eppendorf micro-centrifuge tube and were incubated for 24 h at room temperature. After 24 h, viable phages were enumerated and the numbers of pfu/mL were determined [81].

With regard to temperature, the following temperatures were used: (i) temperatures −20 °C, 4 °C, and 25 °C; (ii) heating the phage at 45 °C, 55 °C, 65 °C, and 75 °C for 15 min and 30 min; (iii) boiling at 100 °C for 15 and 30 min [81]. The viable phages were enumerated and the numbers of pfu/mL were determined [81]. 

The effect of common laboratory disinfectants (ethanol 70%, commercial disinfectant 20% as suggested by the manufacturer, and sodium hypochlorite 2%) on the stability of the phage Ec_MI-02 was determined using LBB-grown preparations of Ec_MI-02. Aliquots (0.1 mL) of the phage (10^7^/mL) were added to 0.9 mL of each disinfectant at the specified concentration in distilled water [81]. After 30, 60, and 120 s incubation at room temperature, the mixtures were assayed for viable phage enumeration (pfu/mL). The control used was a similarly diluted Ec_MI-02 phage, but was untreated with the selected chemical treatments [81]. 

### 4.7. Adsorption Time, Adsorption Rate Constant, Latent Period, and Burst Size of the Phage Ec_MI-02

The adsorption time and the adsorption rate constant of the phage Ec_MI-02 was determined by measuring the residual plaque-forming ability in membrane-filtered samples of an attachment mixture as described by Dowding [82]. Briefly, a 250 mL Erlenmeyer flask containing 50 mL of LBB was inoculated with a host suspension (10^7^ cfu/mL) and was incubated with shaking at 100 rpm for 3 h at 37 °C. The phage was added at multiplicity of infection (0.1) [82], and the flasks were incubated at 37 °C. At various times, samples (1 mL) were removed, membrane filtered, diluted and plated, and the numbers of pfu/mL were counted [82]. 

The ratio of the number of phages to the host was termed the multiplicity of infection and values much less than one are commonly used in kinetic studies on phage growth in order to ensure that each cell is infected by a single virus [83].

The adsorption rate constant, K mL/min, was calculated as described by Sykes et al. [83] using the equation K = 2.3/*Bt* × log _10_ (*P*_0_/*Pt*) where *B* is the host concentration (cfu/mL); *P*_0_ is the initial phage concentration (pfu/mL); *Pt* is the phage concentration at *t* min (pfu/mL); and *t* is the period of adsorption.

In order to determine the latent period, rise period, and the burst size for the phage Ec_MI-02, a one-step growth experiment was carried out as described by Dowding [82]. A suspension (1 × 10^6^ cfu/mL) of the propagation bacterial host was incubated in LBB for 3 h. A predetermined quantity of the phage was then added to give a low multiplicity of infection (approximately 0.1) and incubated for 20 min. An aliquots (10 mL) sample of the attachment mixture was removed and membrane filtered. Unadsorbed phages were removed from the infected host cells by passing the 10 mL of the broth through the filter paper to wash them. The filter paper was then transferred to a flask containing 50 mL of LBB at 37 °C (first growth flask) and the infected cells were re-suspended by agitating the flask. A 50-fold dilution was made to another two flasks held at 37 °C (second and third growth flasks) and the two flasks were re-incubated at 37 °C [82]. Samples (1 mL) were taken (from the first growth flask until time 60 min and then alternately from the second growth flask until 120 min and from the third flask until 180 min), filtered immediately, diluted and plated, and the numbers of pfu were determined after incubating the plates at 37 °C for 48 h [82]. The one-step growth curve for the phage was plotted between pfu/mL and time in min.

### 4.8. TEM of the Phage Ec_MI-02

To determine phage Ec_MI-02 morphology, TEM was used with a negative staining method. Staining with uranyl acetate (Sigma-Aldrich Chemie GmbH, Taufkirchen, Germany) was recommended by Ackermann and Heldal [84] and Burm and Steward [85]. Briefly, solutions containing uranyl acetate were filtered using sterile Millipore membrane syringe filters (0.22 µm). This solution was then placed in a 2 mL screw-cap tube. A drop of the phage suspension (10^10^ pfu/mL) was placed on a 200-mesh copper grid with carbon-coated Formvar film and the excess was removed. A drop of the filtered stain was placed on the grid, which was loaded by the phage Ec_MI-02. After 5 min, the excess stain was removed and allowed to dry for approximately 1 h. Dry grids were stored in a desiccator until inspection. Grids were investigated using a TEM (FEI Bio Twin Spirit G2 TEM, Eindhoven, The Netherlands). 

### 4.9. Isolation of DNA of the Phage Ec_MI-02

Ec_MI-02 was propagated overnight in LBB to reach a titer of 10^8^–10^9^ pfu/mL as described previously [62]. One mL of the suspension was used for DNA isolation using a phage DNA isolation kit (Norgen Biotek Corp, Thorold, ON, Canada), following the manufacturer’s protocol. The extracted DNA was stored at −20 °C for 48 h. 

### 4.10. Genome Sequencing and Assembly of the Phage Ec_MI-02

Samples were sent to Novagen (25 Pandan Crescent #05-15 TIC Centre, Singapore 128477) for whole genome sequencing. The DNA sequencing library was prepared using a NEBNext^®^ Ultra IIDNA Library Prep Kit and sequenced on the Illumina Novaseq 6000 platform. A total of 15,456,744 paired-end reads of 150 bp length were obtained. FastQC v0.11.5 was used to evaluate the quality of the reads [86,87]. BBDuk v38.84 was used to remove adapters and low-quality reads with a minimum quality score of Q30. Furthermore, reads less than 50 bp in length were discarded. As a large number of reads were generated, they was randomly subsampled into three sets of 200,000 paired-end reads each [86].

Geneious assembler in Geneious Prime v2022.2.2 (Geneious Prime 2022.2.2, https://www.geneious.com), was used to independently assemble these de novo. Rapid Annotation using Subsystem Technology (RAST) (https://rast.nmpdr.org; accessed on 2 November 2022), the online prokaryotic genome annotation service, was used to identify open reading frames (ORFs) and to annotate the genome. Protein sequences were manually cross-checked with BLASTP searches (https://blast.ncbi.nlm.nih.gov; accessed on 4 November 2022). 

### 4.11. Bioinformatics Analysis of the Phage Ec_MI-02

To identify phages with most similar genomes to the genome sequence of Ec_MI-02, a nucleotide BLAST (BLASTN) search was performed. ProgressiveMauve was used to compare the whole genome of Ec_MI-02 with the three most identical phages genomes identified in the above step [87]. Terminase large subunit (*TerL*) is commonly used as a genetic marker for the order Caudovirales. *TerL* gene sequence of Ec_MI-02 was compared with sequences of the 15 most identical *TerL* sequences identified by BLASTN. The 16 sequences were aligned using MUSCLE in MEGA 11 [88] (https://www.megasoftware.net; accessed on 27 October 2022). Subsequently, a phylogenetic tree was constructed using these sequences with the maximum likelihood method and 100 bootstraps. 

ABRicate (https://github.com/tseemann/abricate, accessed on 10 January 2023) [64], which employs multiple databases, was used to detect the presence of antibiotic resistance and virulence genes in the Ec_MI-02 genome. HostPhinder [63] was used to predict the host range of Ec_MI-02. Proksee (https://proksee.ca/; accessed on 5 January 2023) was used to visualize the genome map.

### 4.12. Statistical Analysis

All data were analyzed using the analysis of variance (ANOVA) method in SAS Software version 9 (SAS Institute Inc., Cary, NC, USA). Mean values of treatments (four replicates) were compared using Fisher’s protected least significant difference (LSD) test at *p* = 0.05 levels. 

## Figures and Tables

**Table 1:** Host range of the phage Ec_MI-02 using a spot test.

Bacterial Strains	Sensitivity to Phage Ec_MI-02
*Escherichia coli* O157:H7 NCTC 12900	++
*Escherichia coli* O157:H7 (Patient isolate 1)	++
*Escherichia coli* O157:H7 (Patient isolate 2)	++
*Escherichia coli* O157:H7 (Patient isolate 3)	++
*Escherichia coli* O157:H7 (Green salad)	++
*Escherichia coli* O157:H7 (Ground beef)	++
*Escherichia coli* ATCC 8739	++
*Escherichia coli* ATCC 25922	−
*Escherichia coli* ATCC 35218	+
*Escherichia coli* ATCC NCTC 13841	+
*Escherichia coli* ATCC 15223	+
*Escherichia coli* ATCC 23227	+
*Escherichia coli* ATCC 9637	+
*Escherichia coli* ATCC 23224	+
*Escherichia coli* (Patient isolate—urine)	+
*Escherichia coli* ESBL-producing (Patient isolate—blood)	−
*Bacillus subtilis* ATCC 6051	−
*Pseudomonas aeruginosa* ATCC 25668	−
*Pseudomonas aeruginosa* ATCC 27853	−
Methicillin-resistant *Staphylococcus aureus* (Patient isolate)	−
*Staphylococcus aureus* ATCC 6358	−
*Staphylococcus aureus* ATCC 29213	−
*Staphylococcus epidermidis* ATCC 12228	−
*Staphylococcus saprophyticus* ATCC-BAA 750	−
*Streptococcus pyogenes* ATCC 19615	−
*Enterococcus faecalis* ATCC 51299	−
*Enterococcus faecalis* (Patient isolate)	−
*Enterococcus casseliflavus* (Patient isolate)	−
*Enterobacter aerogenes* ATCC 13018	−
*Enterobacter hormaechei* (Patient isolate)	−
*Klebsiella pneumonia* ESBL-producing ATCC 700603	−
*Klebsiella pneumonia* KPC 2 +ve (Patient isolate)	−
*Haemophilus influenzae* ATCC 9007	−
*Stenotrophomonas maltophilia* ATCC 17666	−
*Salmonella enterica* ATCC 14028	−
*Salmonella* sp. (Patient isolate)	−
*Proteus vulgaris* ATCC 29905	−
*Mycobacterium smegmatis* ATCC 607	−

NCTC: national collection of type cultures; ATCC: American type culture collection; ESBL: extended-spectrum beta-lactamase. (++) = host species susceptible to phage lysis with clear plaque, (+) = host species susceptible to phage lysis with turbid plaque, and (–) indicates no plaque. *Escherichia coli* O157:H7 NCTC 12900 was used as the propagation host.

**Table 2:** Host range of the phage Ec_MI-02 using an efficiency of plating (EOP) test.

Bacterial Strains	EOP
*Escherichia coli* O157:H7 (patient isolate 1)	High production (0.86)
*Escherichia coli* O157:H7 (patient isolate 2)	High production (0.9)
*Escherichia coli* O157:H7 (patient isolate 3)	High production (0.89)
*Escherichia coli* O157:H7 (green salad)	High production (0.89)
*Escherichia coli* O157:H7 (ground beef)	High production (0.9)
*Escherichia coli* ATCC 8739	High production (0.6)
*Escherichia coli* ATCC 35218	No production (no plaques)
*Escherichia coli* ATCC NCTC 13841	Low production (0.006)
*Escherichia coli* ATCC 15223	Low production (0.03)
*Escherichia coli* ATCC 23227	Low production (0.03)
*Escherichia coli* ATCC 9637	Low production (0.03)
*Escherichia coli* ATCC 23224	No Production (no plaques)
*Escherichia coli* (Patient isolate—urine)	No production (0.00002)

*Escherichia coli* O157:H7 NCTC 12900 (Original host cell 2.9 × 10^6^ plaque forming units pfu/mL. High production = EOP ≥ 0.5; low production = (0.001< EOP < 0.1); and no production of plaques, (EOP ≤ 0.001).

**Table 3:** Stability of the phage Ec_MI-02 at different pH values.

pH	Initial Titer (IT)	3	4	7	9	10
log_10_ pfu/mL	5.85 ± 0.12 ^a^	5.93 ± 0.048 ^a^	5.96 ± 0.047 ^a^	5.91 ± 0.10 ^a^	5.89 ± 0.10 ^a^	5.92 ± 0.13 ^a^

Values of the number of phages (log_10_ pfu/mL) are means of four independent replicates ± standard deviation. Values with the same letters are not significantly (*p* > 0.05) different within rows, according to Fisher’s Protected LSD Test. pfu, plaque forming units; IT, initial titer ‘’untreated control’’. *Escherichia coli* O157:H7 NCTC 12900 was used as the propagation host.

**Table 4:** Stability of the phage Ec_MI-02 at different temperatures.

	(log_10_ pfu/mL)								
Temperature/Time	Initial Titer (IT)	−20 °C	4 °C	25 °C	45 °C	55 °C	65 °C	75 °C	100 °C
15 min	5.76 ± 0.082 ^Aa^	5.71 ± 0.023 ^Aa^	5.73 ± 0.054 ^Aa^	5.75 ± 0.078 ^Aa^	5.46 ± 0.055 ^Ab^	5.23 ± 0.087 ^Ac^	4.91 ± 0.080 ^Ad^	0.00 ± 0.00 ^Ae^	0.00 ± 0.00 ^Ae^
30 min	5.55 ± 0.078 ^Aa^	5.57 ± 0.043 ^Aa^	5.59 ± 0.056 ^Aa^	5.61 ± 0.055 ^Aa^	5.43 ± 0.032 ^Ab^	5.19 ± 0.114 ^Ac^	4.85 ± 0.067 ^Ad^	0.00 ± 0.00 ^Ae^	0.00 ± 0.00 ^Ae^

Values of the number of phages (log_10_ pfu/mL) are means of four independent replicates ± standard deviation. Values with the same lower-case letters are not significantly (*p* > 0.05) different within rows according to Fisher’s Protected LSD Test. Values with the same upper-case letters are not significantly (*p* > 0.05) different within columns according to Fisher’s Protected LSD Test. pfu, plaque forming units; IT, initial titer “untreated control”. *Escherichia coli* O157:H7 NCTC 12900 was used as the propagation host.

**Table 5:** The effects of chemical disinfectants on the phage Ec_MI-02.

Disinfectant	Initial Titer (IT) (log_10_ pfu/mL)	After 30 s (log_10_ pfu/mL)	After 60 s (log_10_ pfu/mL)	After 120 s (log_10_ pfu/mL)
Ethanol 70%	6.19 ± 0.028 ^Aa^	6.16 ± 0.060 ^Aa^	5.96 ± 0.134 ^Ab^	5.91 ± 0.155 ^Ab^
Commercial disinfectant 20%	6.20 ± 0.057 ^Aa^	5.44 ± 0.121 ^Bb^	4.75 ± 0.149 ^Bc^	3.14 ± 0.240 ^Bd^
Sodium hypochlorite 2%	6.18 ± 0.075 ^Aa^	0.00 ± 0.00 ^Cb^	0.00% ± 0.00 ^Cb^	0.00% ± 0.00 ^Cb^

Values of the number of phages (log_10_ pfu/mL) are means of four independent replicates ± standard deviation. Values with the same lower-case letters are not significantly (*p* > 0.05) different within rows according to Fisher’s Protected LSD Test. Values with the same upper-case letters are not significantly (*p* > 0.05) different within columns according to Fisher’s Protected LSD Test. pfu, plaque forming units; IT, initial titer ‘’untreated control’’. *Escherichia coli* O157:H7 NCTC 12900 was used as the propagation host.

**Table 6:** Ten phage genomes most identical to the genome of the phage Ec_MI-02 as identified by an NCBI BLASTN search (accessed on 27 October 2022).

Bacteriophage	E-Value	Query Coverage	Percentage Identity	Genome Size (bp)	NCBI Accession
*Shigella* phage pSs-1	0.0	97%	98.56%	164,999	NC_025829.1
*Shigella* phage Sfk20	0.0	96%	98.44%	164,878	MW341595.1
*Shigella* phage KNP5	0.0	97%	98.27%	193,624	NC_055713.1
*Escherichia* phage PEC04	0.0	96%	97.57%	167,552	KR233165.1
*Escherichia* phage HY01	0.0	96%	97.57%	166,977	KF925357.1
*Escherichia* phage HY03	0.0	91%	97.38%	170,770	KR269718.1
*Escherichia* phage vB_EcoM_IME537	0.0	95%	97.18%	168,642	NC_054921.1
*Escherichia* phage vB_vPM_PD112	0.0	93%	97.07%	168,084	NC_054928.1
*Escherichia* phage vB_Eco_NR1	0.0	92%	96.95%	167,153	LR990704.1
*Escherichia* phage vB_EcoM_CE1	0.0	94%	96.93%	167,955	ON229909.1

## Data Availability

The complete genome sequence has been deposited in NCBI GenBank under the accession number OP856590.

## Supplementary Materials

The supporting information can be downloaded at: https://www.mdpi.com/article/10.3390/ijms24119506/s1.
