# Supplementary material for: Isolation, Characterization, and Comparative Genomic Analysis of Bacteriophage Ec_MI-02 from Pigeon Feces Infecting Escherichia coli O157:H7
Source: Int J Mol Sci. 2023 May 30;24(11):9506. doi: 10.3390/ijms24119506 (PMC10253784; doi:10.3390/ijms24119506)
Supplement: Supplementary file 1 [file ijms-24-09506-s001.zip › ijms-2382554-supplementary.pdf]

Isolation, Characterization, and Comparative Genomic Analysis of Bacteriophage Ec_MI-02 from Pigeon Feces Infecting *Escherichia coli* O157:H7

Mohamad Ismail Sultan-Alolama [1,2], Amr Amin [2], Ranjit Vijayan [2,3,4,*] and Khaled A. El-Tarabily [2,5,6,*]

[1] Zayed Complex for Herbal Research and Traditional Medicine, Research and Innovation Center, Department of Health, Abu Dhabi 5674, United Arab Emirates
[2] Department of Biology, College of Science, United Arab Emirates University, Al Ain P.O. Box 15551, United Arab Emirates
[3] The Big Data Analytics Center, United Arab Emirates University, Al Ain P.O. Box 15551, United Arab Emirates
[4] Zayed Center for Health Sciences, United Arab Emirates University, Al Ain P.O. Box 17666, United Arab Emirates
[5] Khalifa Center for Genetic Engineering and Biotechnology, United Arab Emirates University, Al Ain P.O. Box 15551, United Arab Emirates
[6] Harry Butler Institute, Murdoch University, Murdoch, WA 6150, Australia
* Correspondence: ranjit.v@uaeu.ac.ae (R.V.); ktarabily@uaeu.ac.ae (K.A.E.-T.)

SUPPLEMENTARY INFORMATION

Table S1: Host range of the two bacteriophages Ec_MI-02 and UAE_MI-01 isolated from pigeon feces against different gram-positive and gram-negative bacteria.

Bacterial strains	Bacteriophage Ec_MI-02 (current study)	Bacteriophage UAE_MI-01 (Sultan-Alolama et al., 2022) [62]
Escherichia coli O157:H7 NCTC 12900	++	++
Escherichia coli ATCC 25922	–	–
Escherichia coli ATCC 8739	++	++
Escherichia coli ATCC 35218	+	–
Escherichia coli ATCC 15223	+	++
Escherichia coli ATCC 23227	+	–
Escherichia coli ATCC 9637	+	–
Escherichia coli ATCC 23224	+	–
Escherichia coli (patient isolate-urine)	+	–
Escherichia coli ESBL-producing (patient isolate-blood)	–	-
Bacillus subtilis ATCC 6051	–	–
Pseudomonas aeruginosa ATCC 25668	–	–
Pseudomonas aeruginosa ATCC 27853	–	–
Methicillin-resistant *Staphylococcus aureus* (patient isolate)	–	–
Staphylococcus aureus ATCC 6358	–	–
Staphylococcus aureus ATCC 29213	–	–
Staphylococcus epidermidis ATCC 12228	–	–
Staphylococcus saprophyticus ATCC-BAA 750	–	–
Streptococcus pyogenes ATCC 19615	–	–
Enterococcus faecalis ATCC 51299	–	–
Enterococcus faecalis (Patient isolate)	–	–
Enterococcus casseliflavus (Patient isolate)	–	–
Enterobacter aerogenes ATCC 13018	–	–
Enterobacter hormaechei (patient isolate)	–	–
Klebsiella pneumonia ESBL-producing ATCC 700603	–	–
Klebsiella pneumonia KPC 2 +ve (patient isolate)	–	–
Haemophilus influenzae ATCC 9007	–	–
Stenotrophomonas maltophilia ATCC 17666	–	–
Salmonella enterica ATCC 14028	–	–
Salmonella sp. (patient isolate)	–	–
Proteus vulgaris ATCC 29905	–	–
Mycobacterium smegmatis ATCC 607	–	–

NCTC: national collection of type cultures; ATCC: American type culture collection; ESBL: extended-spectrum beta-lactamase. (++) = host species susceptible to phage lysis with clear plaque, (+) = host species susceptible to phage lysis with turbid plaque, and (–) indicates no plaque. *Escherichia coli* O157:H7 NCTC 12900 was used as the propagation host.

Table S2: Effect of different pH values on the viability of the two bacteriophages Ec_MI-02 and UAE_MI-01 isolated from pigeon feces.

**pH Value**	**Log_{10} pfu/mL of bacteriophage Ec_MI-02 (current study)**	**Log_{10} pfu/mL of bacteriophage UAE_MI-01 (Sultan-Alolama et al., 2022) [62]**
3	5.93 ± 0.048 aA	0.00 ± 0.00 aB
4	5.96 ± 0.047 aA	5.72 ± 0.12 bB
7	5.91 ± 0.10 aA	7.79 ± 0.11 cB
9	5.89 ± 0.10 aA	7.76 ± 0.15 cB
10	5.92 ± 0.13 aA	7.78 ± 0.16 cB

Values of the number of phages (log_{10} pfu/mL) are means of four independent replicates ± standard deviation. Values with the same lower-case letters are not significantly (p > 0.05) different within columns according to Fisher's Protected LSD Test. Values with the same upper-case letters are not significantly (p > 0.05) different within rows according to Fisher's Protected LSD Test. pfu = plaque forming units. *Escherichia coli* O157:H7 NCTC 12900 was used as the propagation host.

Table S3: Effect of different temperatures on the viability of the two bacteriophages Ec_MI-02 and UAE_MI-01 isolated from pigeon feces.

Temperature	Log_{10} pfu/mL of bacteriophage Ec_MI-02 (current study)	Log_{10} pfu/mL of bacteriophage UAE_MI-01 (Sultan-Alolama et al., 2022) [62]
25 °C for 15 minutes	5.75 ± 0.078 aA	7.46 ± 0.13 aB
25 °C for 30 minutes	5.61 ± 0.055 aB	7.42 ± 0.15 aB
45 °C for 15 minutes	5.46 ± 0.055 bA	7.49 ± 0.14 aB
45 °C for 30 minutes	5.43 ± 0.032 bA	7.48 ± 0.21 aB
55 °C for 15 minutes	5.23 ± 0.087 cA	7.43 ± 0.18 aB
55 °C for 30 minutes	5.19 ± 0.114 cA	7.46 ± 0.12 aB
65 °C for 15 minutes	4.91 ± 0.080 dA	7.47 ± 0.19 aB
65 °C for 30 minutes	4.85 ± 0.067 dA	7.43 ± 0.26 aB
75 °C for 15 minutes	0.00 ± 0.00 eA	3.29 ± 0.09 bB
75 °C for 30 minutes	0.00 ± 0.00 eA	0.00 ± 0.00 bA
100 °C for 15 minutes	0.00 ± 0.00 eA	0.00 ± 0.00 cA
100 °C for 30 minutes	0.00 ± 0.00 eA	0.00 ± 0.00 bA

Values of the number of phages (log_{10} pfu/mL) are means of four independent replicates ± standard deviation. Values with the same lower-case letters are not significantly ($p > 0.05$) different within columns according to Fisher's Protected LSD Test. Values with the same upper-case letters are not significantly ($p > 0.05$) different within rows according to Fisher's Protected LSD Test. pfu = plaque forming units. *Escherichia coli* O157:H7 NCTC 12900 was used as the propagation host.

Table S4: Effect of different common laboratory disinfectants on the viability of the two bacteriophages Ec_MI-02 and UAE_MI-01 isolated from pigeon feces.

Disinfectant	(log_{10} pfu/mL) of bacteriophage Ec_MI-02 (current study)	(log_{10} pfu/mL) of bacteriophage UAE_MI-01 (Sultan-Alolama et al., 2022) [62]
Ethanol 70% Initial Titer	6.19 ± 0.028 aA	7.48 ± 0.11 aB
Ethanol 70% After 2 min	5.91 ± 0.155 bA	6.62 ± 0.06 bB
Sodium hypochlorite 2% Initial Titer	6.18 ± 0.075 aA	7.51 ± 0.15 aB
Sodium hypochlorite 2% After 2 min	0.00% ± 0.00 cA	6.09 ± 0.10 cB
Commercial disinfectant 20% Initial Titer	6.20 ± 0.057 aA	7.45 ± 0.10 aB
Commercial disinfectant 20% After 2 min	3.14 ± 0.240 dA	7.09 ± 0.06 dB
Values of the number of phages (log_{10} pfu/mL) are means of four independent replicates ± standard deviation. Values with the same lower-case letters are not significantly ($p > 0.05$) different within columns according to Fisher's Protected LSD Test. Values with the same upper-case letters are not significantly ($p > 0.05$) different within rows according to Fisher's Protected LSD Test. pfu = plaque forming units. *Escherichia coli* O157:H7 NCTC 12900 was used as the propagation host.		

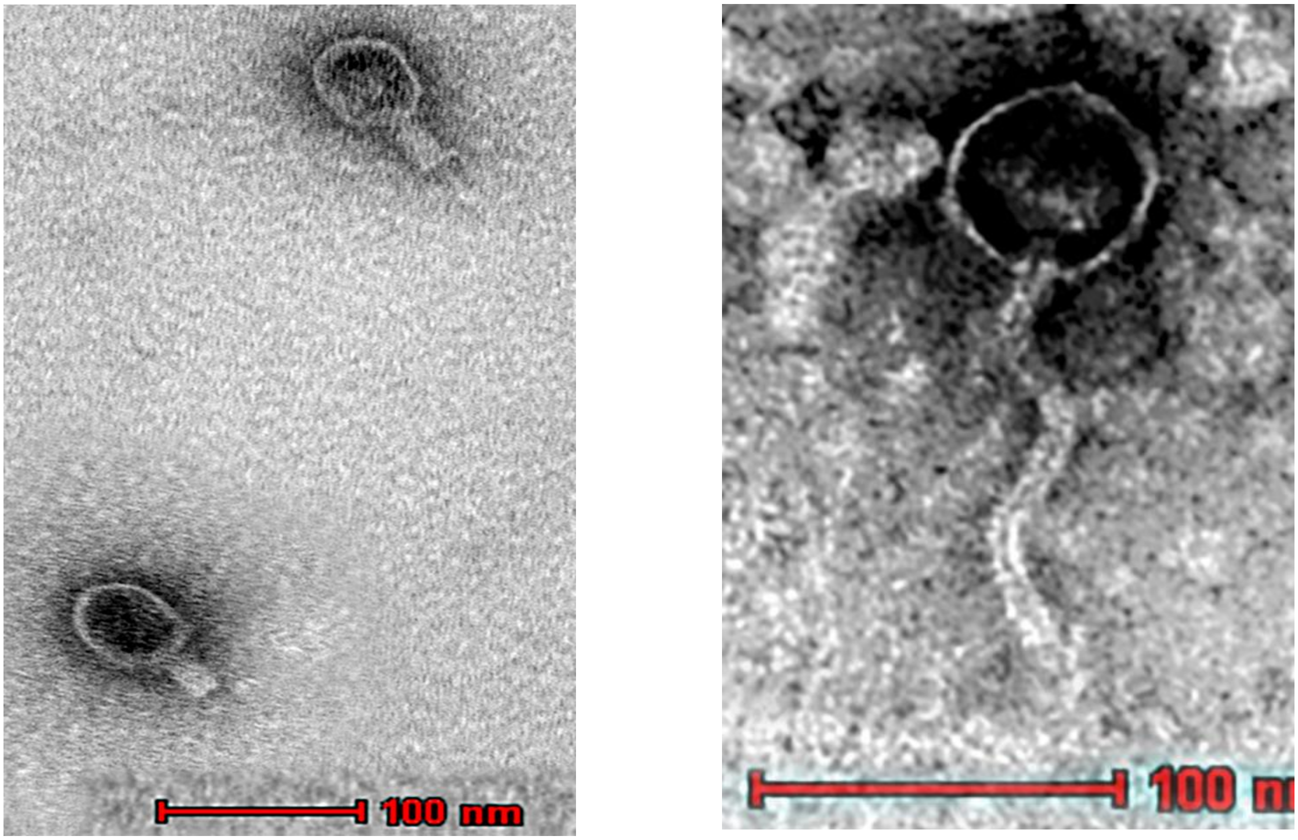

Ec_MI-02

UAE_MI-01

Figure S1. Transmission electron micrograph of the two bacteriophages, Ec_MI-02 (current study, left) and UAE_MI-01 (Sultan-Alolama et al., 2022) [62], right) isolated from pigeon feces Scale bar = 100 nm.

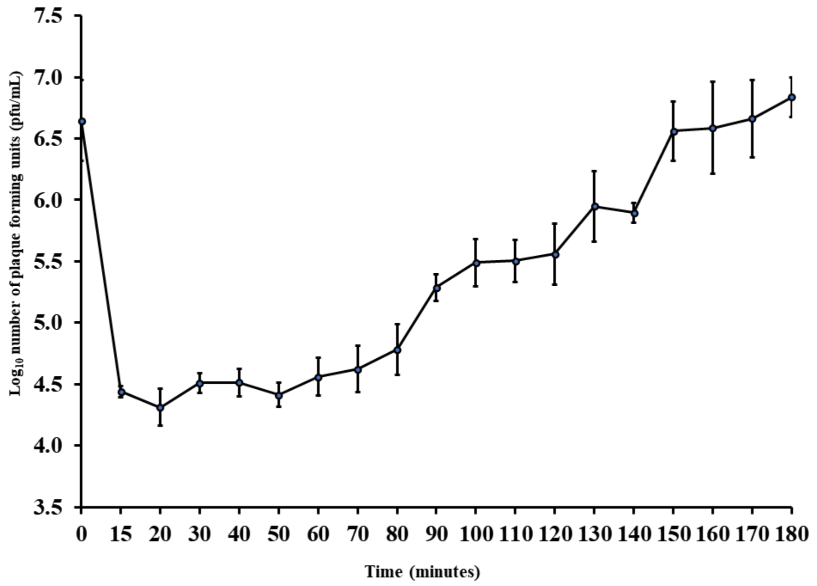

Ec_MI-02

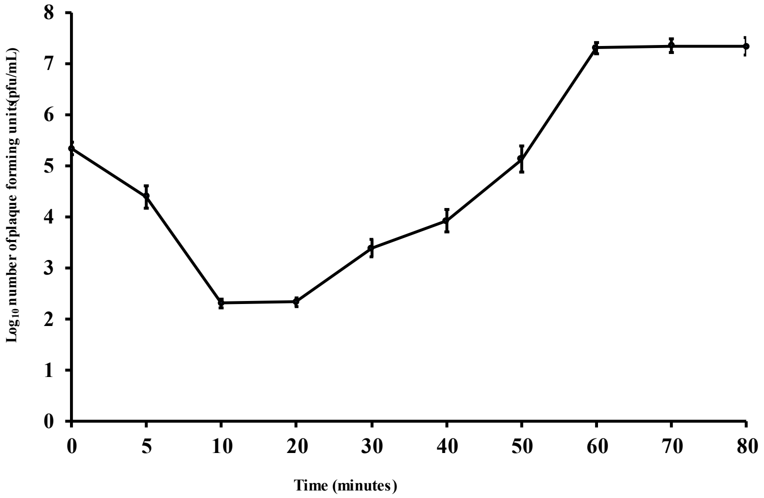

UAE_MI-01

Figure S2. One-step growth curve of the two bacteriophages, Ec_MI-02 (current study, top) and UAE_MI-01 (Sultan-Alolama et al., 2022) [62], bottom) isolated from pigeon feces with *Escherichia coli* O157:H7 NCTC 12900 as the propagation host. Values are means ± standard deviation of four replicates for each timing. Bars represent standard deviation. pfu = plaque forming units.

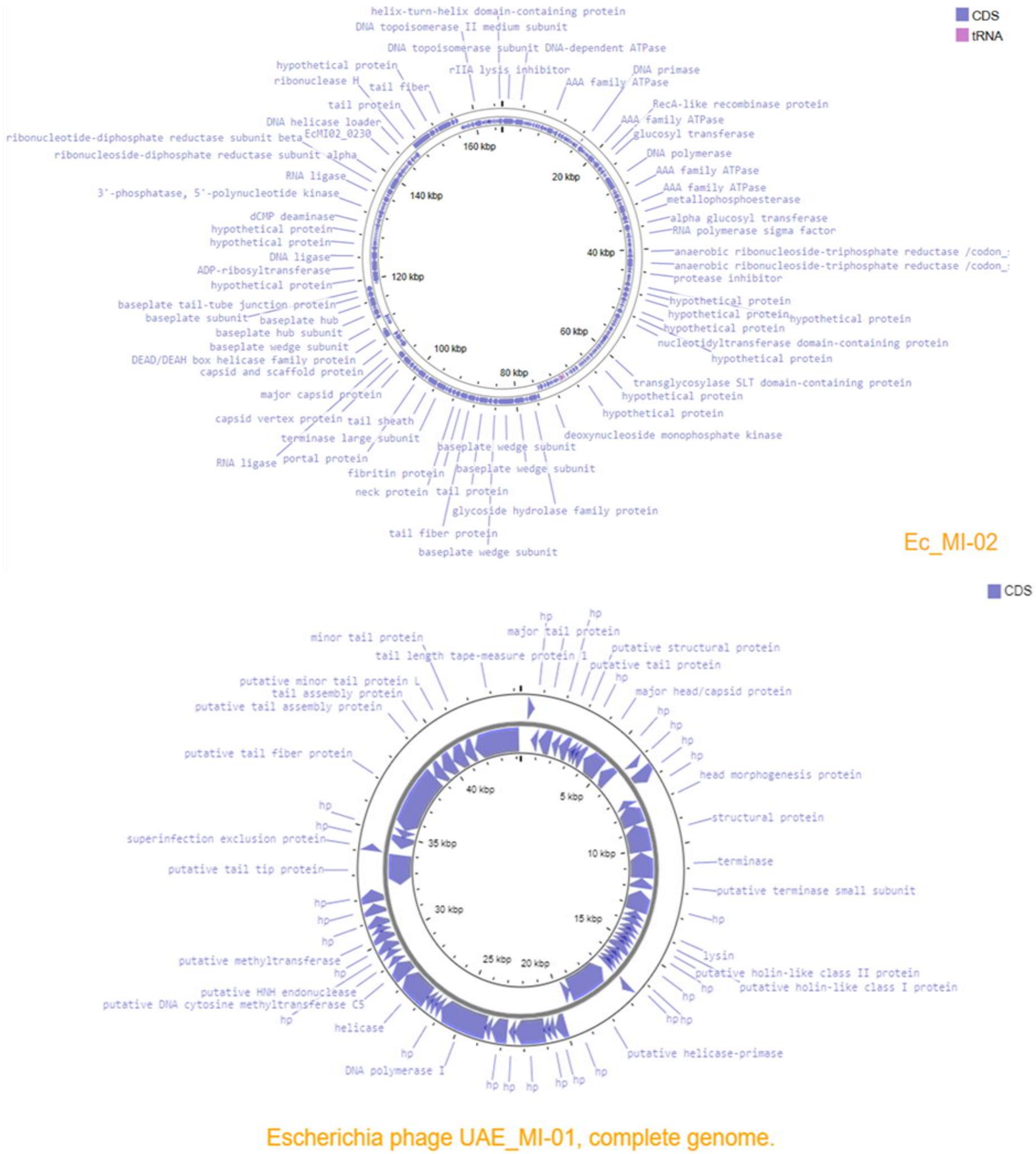

Figure S3. Genome organization map of the two bacteriophages, Ec_MI-02 (current study, top) and UAE_MI-01 (Sultan-Alolama et al., 2022) [62], bottom) isolated from pigeon's feces with *Escherichia coli* O157:H7 NCTC 12900 as the propagation host.

Reference

62. Sultan-Alolama, M.I.; Amin, A.; El-Tarabily, K.A.; Vijayan, R. Characterization and genomic analysis of *Escherichia coli* O157:H7 phage UAE_MI-01 isolated from birds. *Int. J. Mol. Sci*. **2022**, *23*, 14846. doi:10.3390/ijms232314846
